# Dataset of surface water vapour density in southeast, Nigeria

**DOI:** 10.1016/j.dib.2018.02.066

**Published:** 2018-03-07

**Authors:** Sayo A. Akinwumi, Temidayo V. Omotosho, Mojisola R. Usikalu, Oluwole A. Odetunmibi, Oluwafunmilayo O. Ometan, Mustapha O. Adewusi

**Affiliations:** a Department of Physics, Covenant University, Ota, Nigeria; b Department of Mathematics, Covenant University, Ota, Nigeria; c Department of Physics, Lagos state University, Ojo, Lagos state, Nigeria

**Keywords:** Water vapour density, Satellite Communication, Propagation, Atmospheric gases

## Abstract

In this data article, analysis of surface water vapour density in Southeast, Nigeria were reported. The meteorological data were obtained for the period of 39 years between 1973 and 2012 from National Oceanic and Atmospheric Administration (NOAA) Climatology Centre. Five stations considered in the research area includes: Enugu, Onitsha, Abakaliki, Aba and Ihiala. Descriptive statistics were used to show an increase in monthly variation of surface water vapour density (SWVD) minimum value of about 7.15 g/m^3^ at Enugu in January to maximum value of about 21.96 g/m^3^ at Onitsha in April. Hence, the seasonal variation for South East indicate peak value within the months of March to May in the rainy season and a lower value around December to February which is the dry season. The results from this data will help engineers in proper design and planning of radiowave propagation and satellite communication systems in southeastern, Nigeria.

**Specifications Table**

**Table:** 

Subject area	*Meteorology and Atmospheric environment*
More specific subject area	*Satellite Communication, Radiowave propagation, Radio Science*
Type of data	*Table and figure*
How data was acquired	*Secondary data*
Data format	*Raw and analyzed*
Experimental factors	*Data Obtained from National Oceanic and Atmospheric Administration (NOAA) Climatology Centre*
Experimental features	*Computational Analysis: Contingency Tables*
Data source location	*Data Obtained from National Oceanic and Atmospheric Administration (NOAA) Climatology Centre, USAF*
Data accessibility	*All the data are in this article as a supplementary file.*

**Value of the data**

• The data could be useful for government in understanding of radio propagation within or around the lower atmosphere in the southeast region of Nigeria.
• The database could provide insights of surface water vapour density for the five locations.
• The dataset will help engineers in siting good antenna reception at ground level for AM, FM, VHF, UHF bands in Nigeria.
• The data will be useful in understanding of the refractive index structure of the atmosphere through which the waves travel.

## 1 Data

The meteorological data for this article were collected from National Oceanic and Atmospheric Administration (NOAA) Climatology center for the period of about thirty-nine years from 1973 through 2012 for five stations within southeast, Nigeria. The data input parameters such as pressure, temperature, and relative humidity were used for the calculation of surface water vapour density (SWVD) for all the zones. The meteorological data assembled were based on one-minute to produce the daily average data and consequently to acquire the monthly. Therefore, the monthly means of the measurements, over the thirty-nine years is a good characteristic of the seasonal behavior of SWVD as revealed in Table 1a, Table 1b, Table 1c, Table 1d, Table 1e. The descriptive statistics summaries of the SWVD are presented tables. While, bar charts for the SWVD distribution are presented in figures.

**Table 1a:** Monthly Water Vapour Density values from Enugu State.

**Month**	**JAN**	**FEB**	**MAR**	**APR**	**MAY**	**JUN**	**JUL**	**AUG**	**SEP**	**OCT**	**NOV**	**DEC**
**WVD** (g/m^3^)	7.151	14.8	19.588	21.425	20.82	20.196	20.26	20.044	20.617	20.631	16.38	13.66

**Table 1b:** Monthly Water Vapour Density values from Anambra State.

**Month**	**JAN**	**FEB**	**MAR**	**APR**	**MAY**	**JUN**	**JUL**	**AUG**	**SEP**	**OCT**	**NOV**	**DEC**
**WVD (**g/m^3^)	19.8	21.35	21.944	22.01	21.82	20.898	19.526	19.519	20.239	20.591	20.93	19.86

**Table 1c:** Monthly Water Vapour Density values from Ebonyi State.

**Month**	**JAN**	**FEB**	**MAR**	**APR**	**MAY**	**JUN**	**JUL**	**AUG**	**SEP**	**OCT**	**NOV**	**DEC**
**WVD (**g/m^3^)	18.87	20.72	21.241	21.628	21.57	20.698	19.988	19.844	20.124	20.395	20.63	19.12

**Table 1d:** Monthly Water Vapour Density values from Abia State.

**Month**	**JAN**	**FEB**	**MAR**	**APR**	**MAY**	**JUN**	**JUL**	**AUG**	**SEP**	**OCT**	**NOV**	**DEC**
**WVD (**g/m^3^)	19.82	20.69	21.152	21.249	21.17	20.349	19.433	19.144	19.732	19.97	20.43	19.79

**Table 1e:** Monthly Water Vapour Density values from Imo State.

**Month**	**JAN**	**FEB**	**MAR**	**APR**	**MAY**	**JUN**	**JUL**	**AUG**	**SEP**	**OCT**	**NOV**	**DEC**
**WVD (**g/m^3^)	20.62	21.4	21.888	21.961	21.83	20.754	19.703	19.325	20.092	20.511	20.71	20.52

SWVD, g/m^3^, depends on meteorological parameters such as the pressure *P* (mbar), the absolute air temperature *T* (K), and the vapour pressure e (mbar) as given in Eqs. (1), (2), (3):

(1) $$\mathrm{SWVD}=\frac{216.7e}{T}$$

The vapour pressure is also related to the relative humidity *H* (%) as:

(2) $$e=\frac{He_{s}}{100}$$

$e_{s}$ is the maximum (or Saturated) vapour pressure at the given air temperature *t* °C, and may be obtained from:

(3) $$e_{s}=6.11\;\exp[\frac{17.502t)}{(t+240.97)}]$$

The nature and usefulness of the data entails that it can be analyzed using different statistics techniques like descriptive statistics, ordinary least square regression analysis, simple correlation, multiple correlation analysis, analysis of variance, factor analysis and principal component analysis just to mention few.

### 1.1 The summary statistics of the data from Enugu state

**Fig. 1:**
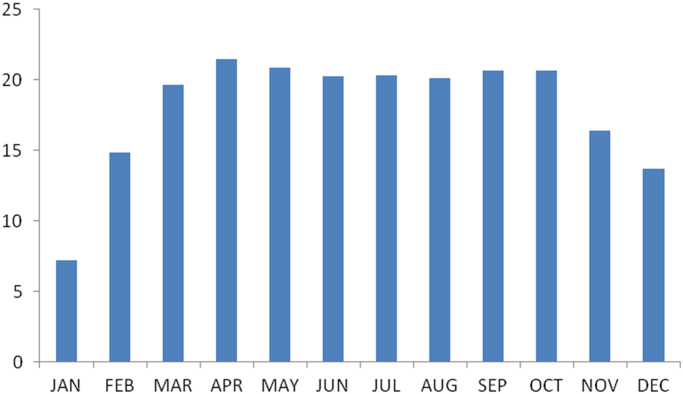
The bar chart showing the monthly water vapor density for Enugu state.

The summary statistics of the data collected from Enugu state is presented in Table 2 below. The data was also presented in a bar chart in Fig. 1. The bar chart is a representation of the descriptive statistics which revealed the level of water vapor density recorded monthly for the state.

**Table 2:** Summary statistics of the Enugu state water vapor density data.

**Statistics**	**Values**
Mean	17.9640
Std. Error of Mean	1.22921
Median	20.1199
Mode	7.15^a^
Std. Deviation	4.25810
Variance	18.131
Skewness	−1.754
Std. Error of Skewness	.637
Kurtosis	2.922
Std. Error of Kurtosis	1.232
Range	14.27
Minimum	7.15
Maximum	21.42

### 1.2 The summary statistics of the data from Anambra state

**Fig. 2:**
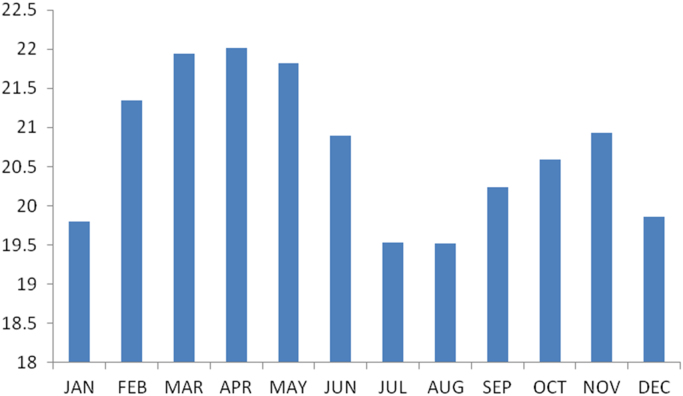
The bar chart showing the monthly water vapour density for Anambra state.

The summary statistics of the data collected from Anambra state is presented in Table 3 below. The data was also presented in a bar chart in Fig. 2. The bar chart is a representation of the descriptive statistics which revealed the level of water vapor density recorded monthly for the state.

**Table 3:** Summary statistics of the Anambra state water vapor density data.

**Statistics**	**Values**
Mean	20.7070
Std. Error of Mean	.26881
Median	20.7449
Mode	19.52^a^
Std. Deviation	.93120
Variance	.867
Skewness	.131
Std. Error of Skewness	.637
Kurtosis	−1.500
Std. Error of Kurtosis	1.232
Range	2.49
Minimum	19.52
Maximum	22.01
Sum	248.48

### 1.3 The summary statistics of the data from Ebonyi state

**Fig. 3:**
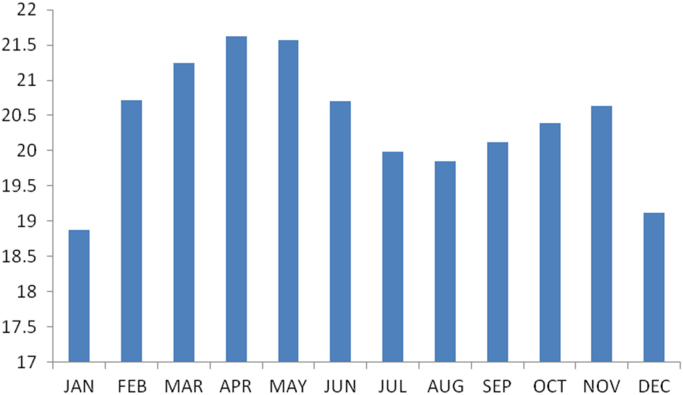
The bar chart showing the monthly water vapor density for Ebonyi state.

The summary statistics of the data collected from Ebonyi state is presented in Table 4 below. The data was also presented in a bar chart in Fig. 3. The bar chart is a representation of the descriptive statistics which revealed the level of water vapor density recorded monthly for the state.

**Table 4:** Summary statistics of the Ebonyi state water vapor density data.

**Statistics**	**Values**
Mean	20.4030
Std. Error of Mean	.25155
Median	20.5131
Mode	18.87^a^
Std. Deviation	.87140
Variance	.759
Skewness	−.305
Std. Error of Skewness	.637
Kurtosis	−.498
Std. Error of Kurtosis	1.232
Range	2.75
Minimum	18.87
Maximum	21.63
Sum	244.84

### 1.4 The summary statistics of the data from Abia state

**Fig. 4:**
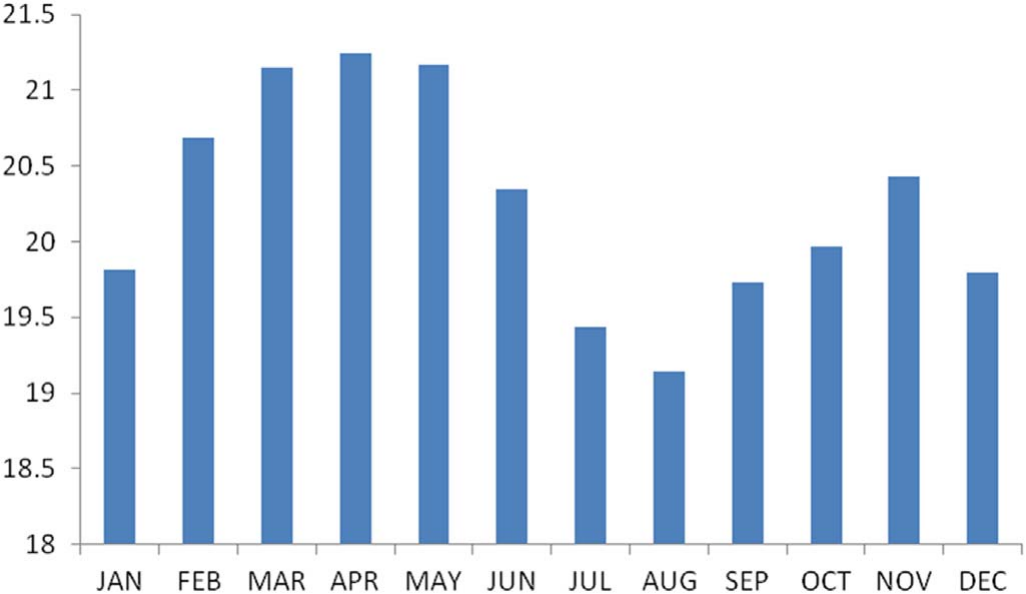
The bar chart showing the monthly water vapor density for Abia state.

The summary statistics of the data collected from Abia state is presented in Table 5 below. The data was also presented in a bar chart in Fig. 4. The bar chart is a representation of the descriptive statistics which revealed the level of water vapor density recorded monthly for the state.

**Table 5:** Summary statistics of the Abia state water vapor density data.

Statistics	Values
Mean	20.2443
Std. Error of Mean	.20471
Median	20.1597
Mode	19.14^a^
Std. Deviation	.70913
Variance	.503
Skewness	.143
Std. Error of Skewness	.637
Kurtosis	−1.211
Std. Error of Kurtosis	1.232
Range	2.11
Minimum	19.14
Maximum	21.25
Sum	242.93

### 1.5 The summary statistics of the data from Imo state

**Fig. 5:**
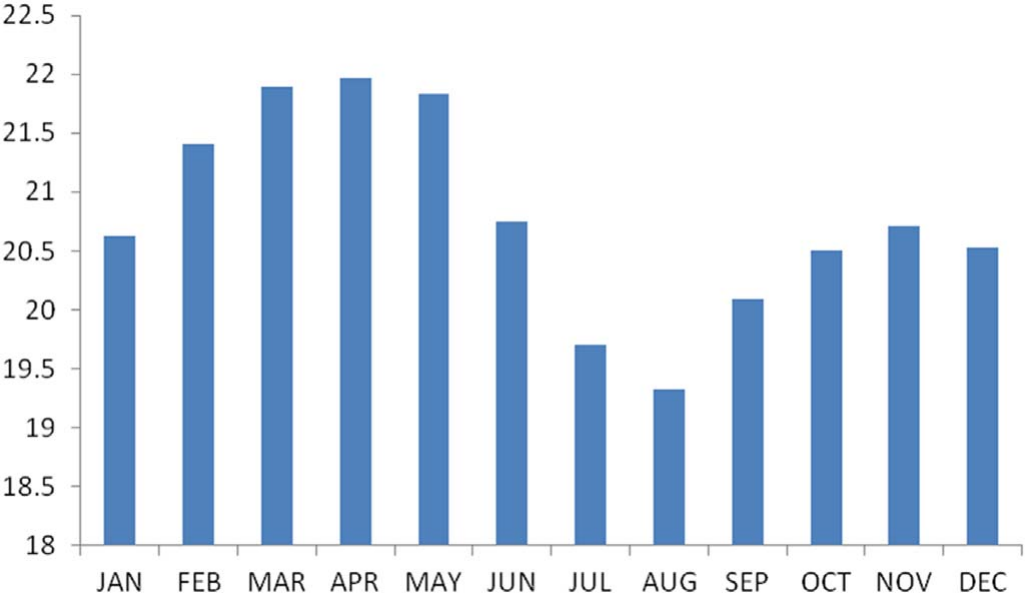
The bar chart showing the monthly water vapor density for Imo state.

The summary statistics of the data collected from Imo state is presented in Table 6 below. The data was also presented in a bar chart in Fig. 5. The bar chart is a representation of the descriptive statistics which revealed the level of water vapor density recorded monthly for the state.

**Table 6:** Summary statistics of the Abia state water vapor density data.

**Statistics**	**Values**
Mean	20.7765
Std. Error of Mean	.24662
Median	20.6655
Mode	19.33^a^
Std. Deviation	.85433
Variance	.730
Skewness	−.037
Std. Error of Skewness	.637
Kurtosis	−.818
Std. Error of Kurtosis	1.232
Range	2.64
Minimum	19.33
Maximum	21.96
Sum	249.32

## 2 Materials and methods

Several researches have been conducted on water vapor density [1], [2], [3], [5], [6], [7], [8], [9], [10], [11], [12], [13], [14], [15], [16]. Similar statistical tools were also applied by [4], [17], [18]. Radiosonde data for at least 39 years between 1973 and 2012 for 5 stations within Southeast Nigeria were utilized for the computation. It was launched from National Oceanic and Atmospheric Administration (NOAA) Climatology center based in United State of America (USA). The variables contained in the meteorology data such as pressure, temperature, and relative humidity were used as input parameters for the outcome of this article.
